# Supplementary material for: How the fear of COVID-19 changed the way we look at human faces
Source: PeerJ. 2021 Apr 29;9:e11380. doi: 10.7717/peerj.11380 (PMC8088764; doi:10.7717/peerj.11380)
Supplement: Supplemental Information 1 [file peerj-09-11380-s001.pdf]

Looking at your eyes? Only if you are COVID-19-free! How the fear of COVID-19 changed the way we look at human faces.

Giovanni Federico, Donatella Ferrante, Francesco Marcatto, Maria Antonella Brandimonte

Post-experimental Interview

1. Demographic information

1.1. Name and surname initials (*free text)*.
1.2. Age (*free text, numbers only*).
1.3. Sex (*f, m, other*).
1.4. City of residence (*free text*).
1.5. Educational level (*none, elementary school, junior high school, high school, bachelor's degree, master's degree, post-graduate/doctorate*).
1.6. Occupation (*free text*).
1.7. Political orientation (*left, centre-left, centre, centre-right, right*).

2. Prior exposure to COVID-19

2.1. I am currently COVID-19 positive (*yes or n*o).
2.2. I got sick of COVID-19, but now I'm cured and negative (*yes or n*o).
2.3. In my circle of acquaintances, someone got sick of COVID-19 (*yes or n*o).
2.4. In my circle of acquaintances, someone has died from COVID-19 (*yes or n*o).

3. Risk perception

Instructions: *"Please answer the following questions with a number ranging from 1 (not at all) to 7 (a lot). There are no right or wrong answers."*

3.1. How risky is it for you coming into contact with a COVID-19 patient?
3.2. How likely is it for you coming into contact with a COVID-19 patient?
3.3. How much can you control the possibility of coming into contact with a COVID-19 patient?
3.4. Are you afraid of coming into contact with a COVID-19 patient?
3.5. Do you know what the consequences of coming into contact with a COVID-19 patient are?
3.6. How serious might be consequences you could have when coming into contact with a COVID-19 patient?

4. COVID-19-related behaviours

Instructions: "*Please indicate how frequently do you do the following behaviours"*.
Possible answers: *NEVER, RARELY, SOMETIMES, OFTEN, VERY OFTEN, ALWAYS*.

When I left home in the last two weeks, I have…

4.1. … used the mask.
4.2. … used gloves.
4.3. … used the hand sanitiser.
4.4. … kept a distance of at least one meter from other people.
4.5. … avoided crowded places.
4.6. … avoided meeting friends/relatives.

5. COVID-19-related intentions

Instructions: "*Please indicate how frequently you will do the following behaviours"*.
Possible answers: *Never, Rarely, Sometimes, Often, Very often, Always*.

When I leave home in the next two weeks, I will…

5.1. … use the mask.
5.2. … use gloves.
5.3. … use the hand sanitiser.
5.4. … keep a distance of at least one meter from other people.
5.5. … avoid crowded places.
5.6. … avoid meeting friends/relative.
