# Supplementary material for: How the fear of COVID-19 changed the way we look at human faces
Source: PeerJ. 2021 Apr 29;9:e11380. doi: 10.7717/peerj.11380 (PMC8088764; doi:10.7717/peerj.11380)
Supplement: Supplemental Information 3 [file peerj-09-11380-s003.pdf]

Questionario post esperimento CVD-19

Gentile partecipante,

Questo questionario costituisce la seconda parte di uno studio di neuroscienze sociali che ha la finalità di studiare alcuni aspetti cognitivi connessi all'attuale situazione pandemica.

Lo studio è stato sviluppato dal Laboratorio di Psicologia Sperimentale dell'Università Suor Orsola Benincasa di Napoli. La partecipazione è su base completamente volontaria.

I tuoi dati personali saranno mantenuti anonimi ed in nessun caso ceduti a terzi.

Per qualsiasi ulteriore informazione, puoi contattare il responsabile della ricerca e della raccolta dati, Dott. Giovanni Federico, inviando un messaggio all'indirizzo di posta elettronica research@giovannifederico.net.

Il tempo previsto per la compilazione del questionario è di circa 15 minuti.

*Campo obbligatorio

Passa alla domanda 1.Passa alla domanda 1.

Informativa privacy

Il/la sottoscritto/a dichiara di essere a conoscenza che:

- ogni partecipante è libero/a di chiedere chiarimenti sulla procedura di raccolta dati e su qualsiasi aspetto dello studio al responsabile della ricerca o a un suo delegato;
- l’attività di ricerca non comporterà alcun pericolo o rischio, temporaneo o permanente, per il benessere psico-fisico dei partecipanti;
- ogni partecipante è libero/a di abbandonare la seduta in qualsiasi fase della ricerca senza alcuna giustificazione;
- il rifiuto a partecipare o l’abbandono alla partecipazione alla ricerca non comporteranno alcuna conseguenza negativa per il partecipante;
- la ricerca è condotta nel rispetto del Codice Etico dell’Associazione Italiana di Psicologia;
- i dati raccolti saranno utilizzati per lo svolgimento della ricerca descritta in premessa, in accordo con le leggi sulla privacy e in conformità al regolamento dell’Unione Europea 679/2016 e il titolare dei dati si impegna a tutelarli secondo tale normativa;
- il titolare dei dati è l’Università Suor Orsola Benincasa di Napoli;
- il responsabile della custodia dei dati, acquisiti in forma anonima e conservati in forma anonima, è il Dott. Giovanni Federico (Università Suor Orsola Benincasa di Napoli) e non sarà consentito ad alcuna persona non autorizzata di accedervi;
- i risultati saranno diffusi in forma aggregata e con ogni cautela necessaria a evitare l’identificabilità dei partecipanti e potranno essere pubblicati su riviste e/o presentati a congressi;

Dichiara inoltre:

- di essere maggiorenne;
- di aver letto con attenzione e compreso compiutamente tutti i punti della precedente dichiarazione;
- di aver potuto porre tutte le domande necessarie al responsabile della ricerca, o a un suo delegato, e aver ottenuto risposte esaurienti;
- di dare il proprio consenso a partecipare alla ricerca;
- di acconsentire al trattamento dei propri dati personali e sensibili raccolti nell’ambito della presente ricerca secondo i termini sopra descritti.

1. Dopo aver letto e compreso l'informativa sulla privacy relativa allo studio... *

Contrassegna solo un ovale.

- ACCETTO di compilare il questionario
- RIFIUTO di compilare il questionario

Il tuo codice

Per mettere in corrispondenza le due parti dello studio garantendoti l'anonimato utilizzeremo un codice.

Il codice si compone in questo modo:

- seconda e terza lettera del nome di tua madre;
- seconda e terza lettera del nome di tuo padre;
- prima lettera del mese in cui è nato tuo padre;
- giorno in cui sei nato/a (se sei nato/a nei primi 9 giorni metti uno 0 prima del numero);

Ad esempio, se mia madre si chiama Giovanna e mio padre Mario ed è nato a marzo e io sono nato il 7 gennaio il mio codice sarà: IOARM07.

2. Inserisci il tuo codice *

____

Informazioni preliminari

Rispondi alle seguenti domande di carattere anagrafico e preliminare.
Ti ricordiamo che i tuoi dati saranno mantenuti anonimi.

3. Iniziali del tuo Nome e Cognome (ad esempio, per Mario Rossi scriverai MR) *

____

4. Età (in anni) *

____

5. Sesso biologico *

Contrassegna solo un ovale.

- ( ) F
- ( ) M
- ( ) Altro: ______________________________

6. Città di residenza *

7. Livello di istruzione *

Contrassegna solo un ovale.

- Dottorato / Master / Specializzazione post laurea
- Laurea specialistica o magistrale
- Laurea triennale
- Scuola media superiore
- Scuola media inferiore
- Scuola elementare
- Nessuno

8. Lavoro svolto

9. Orientamento politico (1 = sinistra; 2 = centro sinistra; 3 = centro; 4 = centro destra; 5 = destra) *

Contrassegna solo un ovale.

	1	2	3	4	5	
Sinistra						Destra

Informazioni relative al COVID-19

10. Sono attualmente COVID-19 positivo *

Contrassegna solo un ovale.

- Sì
- No

11. Mi sono ammalato di COVID-19 ma ora sono guarito e negativo *

Contrassegna solo un ovale.

- Sì
- No

12. Nella mia cerchia di conoscenti, qualcuno si è ammalato di COVID-19 *

Contrassegna solo un ovale.

- Sì
- No

13. Nella mia cerchia di conoscenti, qualcuno è morto a causa del COVID-19

Contrassegna solo un ovale.

- Sì
- No

Percezione del rischio

Rispondi alle seguenti domande con un numero che va da 1 (per nulla) a 7 (tantissimo).
Non esistono risposte giuste o sbagliate.

14. Quanto ritieni sia per te rischioso entrare in contatto con un ammalato di COVID-19? *

Contrassegna solo un ovale.

	1	2	3	4	5	6	7	
Per nulla	◯	◯	◯	◯	◯	◯	◯	Tantissimo

15. Quanto ritieni sia per te probabile entrare in contatto con un ammalato di COVID-19? *

Contrassegna solo un ovale.

	1	2	3	4	5	6	7	
Per nulla	◯	◯	◯	◯	◯	◯	◯	Tantissimo

16. Quanto ritieni di poter controllare la possibilità di entrare in contatto con un ammalato di COVID-19? *

Contrassegna solo un ovale.

	1	2	3	4	5	6	7	
Per nulla	◯	◯	◯	◯	◯	◯	◯	Tantissimo

17. Hai paura di entrare in contatto con un ammalato di COVID-19? *

Contrassegna solo un ovale.

	1	2	3	4	5	6	7	
Per nulla	◯	◯	◯	◯	◯	◯	◯	Tantissimo

18. Sai quali sono le conseguenze dell’entrare in contatto con un ammalato di COVID-19? *

Contrassegna solo un ovale.

	1	2	3	4	5	6	7	
Per nulla	◯	◯	◯	◯	◯	◯	◯	Tantissimo

19. Quanto ritieni possano essere gravi le conseguenze che potresti avere entrando in contatto con un ammalato di COVID-19? *

Contrassegna solo un ovale.

	1	2	3	4	5	6	7	
Per nulla	◯	◯	◯	◯	◯	◯	◯	Tantissimo

Percezione del rischio (2)

Rispondi alle seguenti domande con un numero che va da 1 (per nulla) a 7 (tantissimo).
Non esistono risposte giuste o sbagliate.

20. Quanto ritieni sia per te rischioso entrare in contatto con una persona guarita da COVID-19? *

Contrassegna solo un ovale.

	1	2	3	4	5	6	7	
Per nulla	◯	◯	◯	◯	◯	◯	◯	Tantissimo

21. Quanto ritieni sia per te probabile entrare in contatto con una persona guarita da COVID-19? *

Contrassegna solo un ovale.

	1	2	3	4	5	6	7	
Per nulla	◯	◯	◯	◯	◯	◯	◯	Tantissimo

22. Quanto ritieni di poter controllare la possibilità di entrare in contatto con una persona guarita da COVID-19? *

Contrassegna solo un ovale.

	1	2	3	4	5	6	7	
Per nulla	◯	◯	◯	◯	◯	◯	◯	Tantissimo

23. Hai paura di entrare in contatto con una persona guarita da COVID-19? *

Contrassegna solo un ovale.

	1	2	3	4	5	6	7	
Per nulla	◯	◯	◯	◯	◯	◯	◯	Tantissimo

24. Sai quali sono le conseguenze dell’entrare in contatto con una persona guarita da COVID-19? *

Contrassegna solo un ovale.

	1	2	3	4	5	6	7	
Per nulla	◯	◯	◯	◯	◯	◯	◯	Tantissimo

25. Quanto ritieni possano essere gravi le conseguenze che potresti avere entrando in contatto con una persona guarita da COVID-19? *

Contrassegna solo un ovale.

	1	2	3	4	5	6	7	
Per nulla	◯	◯	◯	◯	◯	◯	◯	Tantissimo

Comportamenti

QUANDO SONO USCITO DA CASA NELLE ULTIME DUE SETTIMANE HO...

26. ...usato la mascherina *

Contrassegna solo un ovale.

- mai
- raramente
- qualche volta
- spesso
- molto spesso
- sempre

27. ...usato i guanti *

Contrassegna solo un ovale.

- mai
- raramente
- qualche volta
- spesso
- molto spesso
- sempre

28. ...usato l'igienizzante *

Contrassegna solo un ovale.

- mai
- raramente
- qualche volta
- spesso
- molto spesso
- sempre

29. ...mantenuto la distanza di almeno un metro dalle altre persone *

Contrassegna solo un ovale.

- mai
- raramente
- qualche volta
- spesso
- molto spesso
- sempre

30. ...evitato i luoghi affollati *

Contrassegna solo un ovale.

- mai
- raramente
- qualche volta
- spesso
- molto spesso
- sempre

31. ...evitato di incontrare amici/parenti *

Contrassegna solo un ovale.

- mai
- raramente
- qualche volta
- spesso
- molto spesso
- sempre

Intenzioni

QUANDO USCIRÒ DA CASA NELLE PROSSIME DUE SETTIMANE HO INTENZIONE DI...

32. ...usare la mascherina *

Contrassegna solo un ovale.

- per nulla probabile
- improbabile
- poco probabile
- probabile
- molto probabile
- altamente probabile

33. ...usare i guanti *

Contrassegna solo un ovale.

- per nulla probabile
- improbabile
- poco probabile
- probabile
- molto probabile
- altamente probabile

34. ...usare l'igienizzante *

Contrassegna solo un ovale.

- per nulla probabile
- improbabile
- poco probabile
- probabile
- molto probabile
- altamente probabile

35. ...mantenere la distanza di almeno un metro dalle altre persone *

Contrassegna solo un ovale.

- per nulla probabile
- improbabile
- poco probabile
- probabile
- molto probabile
- altamente probabile

36. ...evitare i luoghi affollati *

Contrassegna solo un ovale.

- per nulla probabile
- improbabile
- poco probabile
- probabile
- molto probabile
- altamente probabile

37. ...evitare di incontrare amici/parenti *

Contrassegna solo un ovale.

- per nulla probabile
- improbabile
- poco probabile
- probabile
- molto probabile
- altamente probabile

Questi contenuti non sono creati né avallati da Google.

Google Moduli
